# Using Radio-Frequency Identification Technology to Measure Synchronised Ranging of Free-Range Laying Hens

**DOI:** 10.3390/ani8110210

**Published:** 2018-11-16

**Authors:** Dana L.M. Campbell, Brian J. Horton, Geoff N. Hinch

**Affiliations:** 1 Agriculture and Food, CSIRO, New England Highway, Armidale, NSW 2350, Australia; 2 School of Environmental and Rural Science, University of New England, Armidale, NSW 2350, Australia; ghinch@une.edu.au; 3 Tasmanian Institute of Agriculture, School of Land and Food, University of Tasmania, Launceston, TAS 7250, Australia; brian.horton@utas.edu.au

**Keywords:** social patterns, cohesion, group dynamics, early enrichment, RFID, hen movement

## Abstract

**Simple Summary:**

Free-range laying hens can choose to be indoors or outdoors. Individual hens vary in their ranging choice and this behaviour could also be affected by their flock mates. Radio-frequency identification tracking of individual hens in experimental free-range pens with group sizes of 46–50 hens was used to study flock ranging patterns. Across the day, hens moved through the range pop-holes in the same direction as other hens above levels expected by random chance, termed ‘pop-hole-following’. Hens were also simultaneously indoors or outdoors with other specific hens more often than expected by random chance, termed ‘hen-pair association’. Chicks that were provided variable stimulatory and structural enrichments from 4 to 21 days showed higher pop-hole-following and hen-pair association than non-enriched birds. The individual birds within these small hen groups were behaving primarily as a cohesive flock which has implications for understanding the group-level behaviour of hens. Further research would analyse if similar social movement patterns were present in larger commercial free-range flocks and how early rearing environments may affect adult social behaviour.

**Abstract:**

Free-range laying hen systems provide individuals a choice between indoor and outdoor areas where range use may be socially influenced. This study used radio-frequency identification technology to track the ranging of individually-tagged hens housed in six experimental free-range pens from 28 to 38 weeks of age (46–50 hens/pen). All daily visits to the range were used to study group behaviour. Results showed that 67.6% (SD = 5.0%) of all hen movements through the pop-holes outdoors or indoors were following the movement of another hen (‘pop-hole-following’) compared to only 50.5% of movements in simulated random data. The percentage overlap in time that all combinations of hen pairs within each pen spent simultaneously outdoors or indoors showed a median value of overlap greater than the 90th percentile of random data. Pens housing hens that had been provided variable enrichments from 4 to 21 days (n = 3 pens) showed higher ‘pop-hole-following’ behaviour and a higher percentage of hen-pair association compared to hens reared in non-enriched conditions (n = 3 pens). These results show that birds in each free-range pen were primarily a cohesive flock and early enrichment improved this social cohesiveness. These results have implications for understanding free-range flock-level behaviour.

## 1. Introduction

Animals living within groups will typically exhibit both individual behaviour patterns and engage in social interactions resulting in group-level dynamics. Laying hens (*Gallus gallus domesticus*) are a domesticated gregarious species, and with the increase in alternative cage-free housing systems, hens are often kept in groups of thousands of individuals. Natural circadian rhythms dictate typical behavioural patterns for laying hens that comprise egg laying in the morning, dust bathing in the afternoon, and roosting at night [1,2,3]. Behavioural synchrony of a group of intensively-housed animals has been used as both a positive and negative indicator of welfare [4]. In laying hens, flock-level synchrony can lead to overcrowding of preferred resources during specific periods of peak demand [1,2]. Alternatively, synchrony may facilitate resting [5] and flock-level cohesion may also result in an even temporal distribution of birds to optimise use of the available resources. This could include steady transitions between system areas [6] or adjusting egg-laying patterns to ensure nest box availability [3].

In free-range commercial and experimental housing systems, high individual variation in use of the outdoor area is well documented [7,8,9,10], including locational preferences within the indoor part of the system [11]. In experimental free-range settings, different pens of birds housed simultaneously in the same shed environments also show high variation in levels of range access [7,8]. In general, sequential flocks of birds housed in the same environments may respond very differently to their surrounding conditions. Producers comment on this phenomenon, particularly where some management practices work well for some flocks, and for no discernible reason, do not work well for others (pers. comm. to DLMC, 2017). Variation between flocks of the same strain and incubation cohort may be a reflection of parent-stock effects [12]. They could also be a reflection of group dynamics such as fearful or stressed individuals having impacts on their flock-mates through social contagion [13]. The factors that impact the group dynamics are likely to be multimodal, but increased understanding of causative stimuli may improve group-level management. There is currently limited understanding of how individual birds may be interacting on a group level in a free-range system that provides a choice of accessing an outdoor area or not and whether the degree of social influence on ranging varies between different pen groups.

Observations of red junglefowl ancestors in their natural habitat (India), showed small groups (up to seven individuals) comprised of males and females or all-male subgroups within the flocks [14]. Similarly, in semi-wild populations, consistent subgroups were formed within specific territories and dominance hierarchies were observed among individuals of the flock [15]. In experimental conditions, laying hens in groups of 10 individuals are able to discriminate between familiar and unfamiliar individuals, but do not discriminate when group size is 120 individuals, suggesting dominance hierarchies do not form in larger groups [16]. As group size increases, hens may be unable to recognise all individuals and it could be too energetically costly to establish a hierarchy via continual aggressive encounters with unfamiliar birds [16,17]. However, observations of marked birds in a commercial tiered aviary system with group sizes of approximately 500–600 individuals, found hens formed roosting subgroups of approximately 10 birds and maintained close association during daytime localised system use [18].

Further observations from video recordings of small groups (15 hens/group) of individually-marked hens in an experimental floor-pen setting documented dyadic associations during daytime system use and evening roosting [19]. There was some evidence that roosting preferences were not random but overall, no conclusive evidence was found for hens preferentially associating with each other in these small experimental groups [19]. Similarly, limited evidence of social relationships in a group of eight hens was confirmed by [20]. It is currently unknown if range use in free-range hens may be related to social associations between individual birds.

The degree to which individual hens in a group interact socially can be impacted by multiple factors. These may include resource availability and distribution [21], hen strain [22], and flock size [23,24]. The rearing environment of pullets can impact development of socially-stimulated behaviours such as feather pecking [25]. Provision of enrichments during rearing such as litter substrate can reduce the development of feather pecking [26], while the provision of aerial perches for adult free-range hens reduced aggression [27]. However, there is no research on the impacts of enrichment during rearing on the social associations or social influence on ranging in adult free-range laying hens.

In the present study, radio-frequency identification data of individual bird movement in and out of the pop-holes were used to first determine if range access of hens housed in small experimental flocks was socially-influenced by the range access of flock-mates. This was assessed using actual movement patterns against generated random data. Secondly, to determine whether there was overlap in time spent together either indoors or outdoors between individual birds that may indicate hens had social associations and thirdly, whether environmental enrichment during the first 3 weeks of age had impact on these ranging patterns of adult hens.

## 2. Materials and Methods

### 2.1. Ethical Statement

All research was approved by the University of New England Animal Ethics Committee (AEC 15-119).

### 2.2. Chick Housing

Day-old Hy-Line^®^ Brown chicks (n = 290) with an infra-red beak trim were obtained (November 2015) from a commercial supplier (11 birds died across the duration of the trial). All chicks were randomly allocated into two separate rooms (L × W: 4.5 m × 3 m) at the University of New England where they were housed until 12 weeks of age. Heating and hours of light in both rooms followed the Hy-Line^®^ Brown rearing management guide (Hy-Line^®^ International, 2014). Birds were provided commercial mash ad libitum, formulated for specific growth stages, access to water nipples (10 birds/nipple) and wood shavings as a floor substrate. One perch rack (H × W: 1.6 m × 2.2 m with 6 perch bars evenly spaced across the 1.6 m height) per room was added at 4 weeks of age. The birds in the two separate rooms were subject to different rearing treatments from 4 to 21 days of age. These birds were reared for a larger overall study assessing the effects of early enrichment on adult range use, welfare and response to implemented environmental stressors [8]. In the enriched room, birds were provided an array of novel objects and stimuli (including patterned wallpaper, cinder blocks, large sealed plastic tubs, cat and dog toys attached to feeders and water nipples, randomly-scheduled coloured flashing lights and auditory playbacks that included sounds of doors opening, moving vehicles, weather, voices, machinery), that were regularly changed to simulate an unpredictable environment. In the non-enriched conditions, birds had no additional interventions. After 3 weeks, the enrichments were removed and birds within the two rooms were subsequently housed under similar conditions.

### 2.3. Pullet and Layer Housing

At 12 weeks of age, all birds were transported to the University of New England’s experimental free-range facility located in Armidale, Australia. Birds were distributed between 6 indoor pens in alternating treatment order (3 enriched-rearing treatment pens; 3 non-enriched-rearing treatment pens). Birds within treatments were randomly placed into one of three pens, however, a subset of birds had been previously tested in behavioural tests as part of a separate unpublished dataset and these were balanced in placement across treatment pens. There were also 7 additional non-enriched over enriched birds. This resulted in 46–50 birds per pen. The pens all had equal resources (one round feeder, 15 water nipples, one perch rack and 12 nest boxes) that exceeded the Australian Model Code of Practice for the Welfare of Animals—Domestic Poultry [28]. For a detailed schematic of the indoor pen configuration, see [8]. Birds were fed commercial mashes ad libitum, formulated for pullet and then layer life stages. Indoor stocking density was approximately 3 birds/m^2^ and rice hulls were used as a litter substrate. The shed was fan-ventilated but not temperature or humidity controlled.

### 2.4. Outdoor Ranges and Radio-Frequency Identification

**Figure 1:**
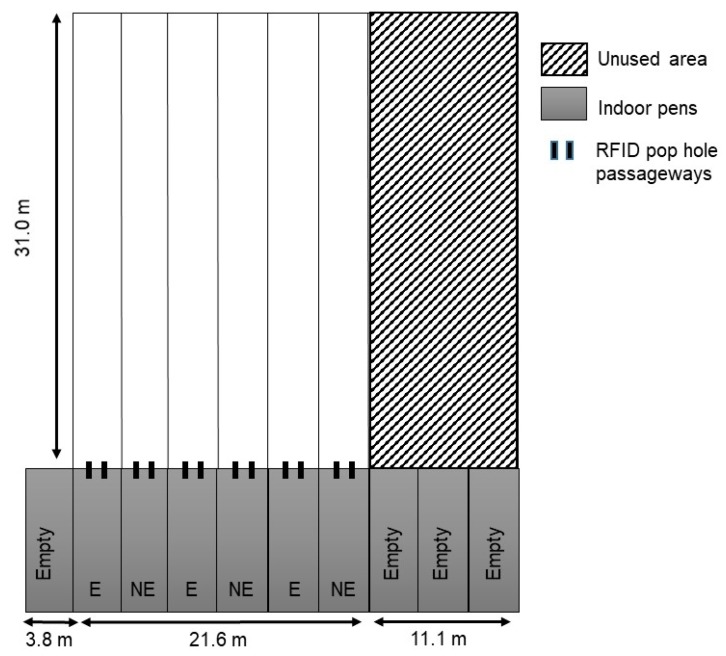
The experimental free-range facility showing the 6 indoor pens connected to separate outdoor range areas and the two radio-frequency identification (RFID) passageways within each pop-hole. Each pen housed birds from either enriched (E) or non-enriched (NE) rearing treatments.

The 6 indoor pens each had a separate fenced (2 m high to prevent birds flying over) straight outdoor run (L × W: 31 m × 3.6 m) with no trees or shelter structures present (outdoor stocking density approximately 4200 hens per hectare, Figure 1). Shade cloth (Universal Shade Cloth, 90% UV block grade, Shade Australia, Ingleburn, NSW, Australia) was placed doubled over at a height of 0.9 m along the fences to restrict visual contact between birds in each separate run. The pop-holes that provided range access were first opened at 21 weeks of age (April 2016) with subsequent daily access from 09:00 to 16:30 h for 17 weeks over autumn and winter. Hens were not forced onto the range at any time and were trained to return inside each afternoon using poultry mixed grain shaken in a metal tin, with pop-holes closed at all other times. Any birds that remained outside in the afternoon following the audio signals of the mixed grain were gently encouraged back inside by a person walking out on the range.

**Figure 2:**
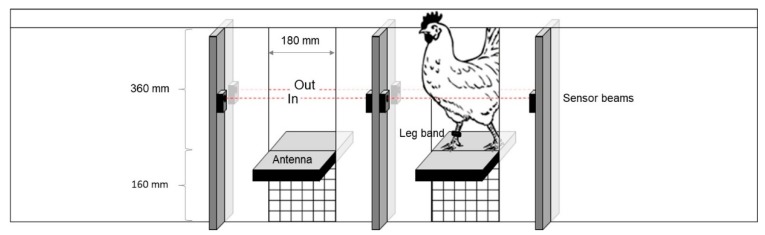
The radio-frequency identification system placed within the pop-holes of each pen. Two passageways allowed the movement of a single bird each across an antenna. Two sets of sensor beams allowed the determination of movement direction, ‘out’ to the range, or back ‘in’.

At 20 weeks of age, all birds were fitted with an adjustable leg band (Roxan Developments Ltd., Selkirk, UK) containing a glued RFID microchip (Trovan^®^ Unique ID 100 (FDX-A): Microchips Australia Pty Ltd., Keysborough, VIC, Australia). RFID systems consisting of two passageways (H × W: 36 cm × 18 cm) designed to allow the passage of only a single bird at any one point in time were placed within the pop-hole frame (Figure 1). The RFID system consisted of 610f IP68 antennas placed within each passageway (Figure 2), connected to Trovan LID 650N decoders with all equipment developed and built by Dorset Identification B.V. (Aalten, The Netherlands) using Trovan^®^ technology. Paired optical sensor beams were placed inside and outside the pop-holes to track the direction of bird movement (onto the range or into the pen; Figure 2) with the antennas reading the leg bands at a precision of 0.024 s (maximum detection velocity 9.3 m/s) and a detection range of up to 18 cm. Dimensions of the pop-hole and placement of the sensors (as per Figure 2) enabled 100% detection rate as visually confirmed by monitoring bird movement and matching with real-time readings displayed on a connected tablet. The date and time of individually-tagged birds passing through and in which direction were recorded with all data downloaded directly to a USB flash drive via a data logger. 

### 2.5. RFID Data Analyses

RFID data were initially run through a custom-designed software program written in the ‘Delphi’ language (Bryce Little, CSIRO, Agriculture and Food, St Lucia, QLD, Australia) that filtered out any unpaired or ‘false’ readings that may occur if, for example, a hen sits inside the pop-hole but does not complete a full transition onto the range or back into the pen. Once screened in this way, the data were used for further analysis.

A 15-day period of ranging from 36 to 38 weeks of age was selected for range use analyses of mean group behaviour using individual-bird data. Comparisons were made between enriched and non-enriched treatment groups including variation between pens within treatment groups. These ages represented the longest time the birds had been ranging prior to implemented environmental stressors as described in [8] that affected range use. The birds had relatively established ranging patterns and the two-week time period minimised any effects of age. The custom-designed software program summarised the daily data to provide a mean value per individual bird for hours outdoors, number of visits outdoors, and mean maximum time for individual visits. The visit count data were square-root transformed, with the raw values displayed in the results as there was virtually no difference between the raw and back-transformed values. General Linear Model analyses that included treatment and pen nested within treatment as fixed effects were conducted on each range use variable using JMP^®^ 13.0 (SAS Institute, Cary, NC, USA) with α set at 0.05. Where significant differences were present, post-hoc Student’s *t*-tests were applied to the least squares means and a Bonferroni correction was applied to multiple comparisons.

Box plots generated by JMP^®^ showed lines within the boxes to represent the median with the lower and upper box boundaries representing the interquartile range (i.e., difference between the 1st and 3rd quartiles). The whiskers were drawn to the outermost data point that fell within the following distances: upper whisker = 3rd quartile + 1.5 × (interquartile range) and lower whisker = 1st quartile − 1.5 × (interquartile range). If the data points did not reach these computed ranges, whiskers were determined by the upper and lower data point values, with disconnected points displaying potential outliers.

### 2.6. Determining Association Patterns Using RFID Data

Individual bird data were then used to describe hen behavioural movement patterns in and out of the pop-holes and inter-hen associations as detailed in the following sections. A period of 60 days from 28 weeks to 38 weeks of age was selected giving the birds several weeks to first adjust to range access (see [8]). Three days within this period were excluded because of an abnormally low or high number of movements on that day. For example, a thunderstorm on one day resulted in half the usual time outside.

#### 2.6.1. Random Data

From the daily individual-bird data, the movement times were measured within each of the 6 pens to provide a single value per hen per day for each of: (1) mean time to go outside after the pop-holes were first opened, (2) mean time spent outside during each individual range visit, (3) mean time spent inside on each return during the day when the pop-holes were open and hens had a choice of location and (4) mean time between the last return inside for the day and 16:30 h, when the pop-holes were closed for the night. Including this last variable allowed for differences between hens that consistently went inside early compared to those that responded to the sound cue of mixed grain in a metal tin. It also provided a method of ensuring each hen was inside at the end of the day. The mean values for each pen are shown in Table 1.

All data were log-transformed to approach normality with the mean and standard deviation based on the log-times. Using the same number of days as used in the recording period, a set of random data were generated. This was a dataset of hen movements, comparable to the actual movements but based on mean values per hen where birds were behaving independently of (at random) the rest of the flock. This was done to determine if actual patterns deviated significantly from a random pattern. It was achieved by each hen being assigned a time when it would next move, based on whether it was currently inside or outside. The time was selected using a random value based on the log-mean and standard deviation for its next movement in the other direction. Sequentially, the next hen to move was found and its next movement time reset and this continued throughout the day until all hens had returned inside by the end of the day (see Supplementary Materials 1 for the generation of the random dataset).

#### 2.6.2. Random Follower Data

A second set of random data of hens following each other through the pop-holes (termed ‘pop-hole-following’ hereafter) was also used to generate a dataset where hens followed another random hen (follower data). This dataset was created because an inspection of the original data suggested that following behaviour was possible where a hen’s next movement was more often in the same direction as the preceding hen’s movement than the opposite direction. Therefore, the generation of data was adjusted to simulate a follower effect, without causing the hens to follow any specific hen. After each movement, all hens whose next movement would be in the opposite direction to that movement had their expected movement time delayed by a short period of 40 s that was determined by using a trial and error process. This time period was determined by increasing or decreasing it by small increments until the percentage of pop-hole-following behaviour in the random data was consistent with that observed in the actual hen movements. This increased the probability that the next movement would be in the same direction as the last movement and the data generated represent hens who did not follow any specific hen on each occasion. These follower data were used to determine if hens were following specific hens.

### 2.7. Associations between Pairs of Hens in Time Together

Using the same daily individual bird data from 28 to 38 weeks of age, for every possible pair of hens, A and B, the time spent together was measured as the average of the following:

(a) Total time when both were outside as a % of the time when A was outside.
- Total time when both were outside as a % of the time when B was outside.
- Total time when both were inside as a % of the time when A was inside.
- Total time when both were inside as a % of the time when B was inside.

These time measures were chosen because if hen A spends almost all of her time outside then she will overlap in time with every other hen, giving a high score for (b). Hen A will also score high for (a) with other hens that also spend the majority of the time outside. However, she will only score high for (c) and (d) if the small amount of time that hen A is inside occurs at the same time as hen B is inside. Therefore, the four measures combine to provide a measure of the degree of overlap in time together for each pair of hens. The time hens spent associated was compared with results from the ‘random’ and ‘follower’ groups of hens, where there were no associations between hens. The consistency of this overlap in hen association over time was examined by comparing results for successive 10-day periods over a total period of 60 days. Pearson’s Product-Moment Correlations between adjacent and non-adjacent periods were assessed using Microsoft Excel.

The effect of early rearing treatment on hen association was also assessed. The percentage association data for the successive 10-day periods for each pen (n = 36: 6 pens × 6 time periods) were converted into proportions and logit transformed. Data were analysed using a General Linear Model in JMP^®^ 13.0, with early enrichment rearing treatment and pen nested within treatment as fixed effects with α set at 0.05. Where significant differences were present, post-hoc Student’s *t*-tests were applied to the least squares means, and a Bonferroni correction was applied to multiple comparisons.

### 2.8. Pop-Hole-Following Scores

Pop-hole-following behaviour was quantified for individual birds using the 15-day period from 36 to 38 weeks of age. This shorter time period limited any impact of age and allowed a focused assessment of hen behaviour when birds would be more likely to have established ranging patterns. The number of movements that were in the same direction as the movement by the previous moving hen, were expressed as a percentage of the total number of movements by that hen. These data were used to assess differences between early enrichment rearing treatments and pens within rearing treatments. Individual-bird pop-hole-following scores were logit transformed and analysed using a General Linear Model, with early enrichment rearing treatment and pen nested within treatment as fixed effects. Analyses were performed in JMP^®^ 13.0, post-hoc Student’s *t*-tests were applied to the least squares means, and α was set at 0.05.

### 2.9. Associations between Pairs of Hens When Following

Finally, the data from the 15-day period from 36 to 38 weeks of age were used to assess for association between hens when following. For all days in this period, for every possible pair of hens, the distance between these hens (i.e., the number of other hens between them) was measured for each occasion when either hen moved in or out. The median ‘distance’ between that pair of hens was a measure of the social distance between them when moving inside or outside. For example, if hen A moved outside, then 10 other hens moved outside before hen B moved outside, then hens A and B were separated by 10 hens. Movements inside were tracked and ordered independently of movements outside. A pair of hens were considered associated if the median distance was less than a set value determined as 8 or 9 hens, values at which associations began to be seen.

## 3. Results

### 3.1. Range Use from 36 to 38 Weeks

**Figure 3:**
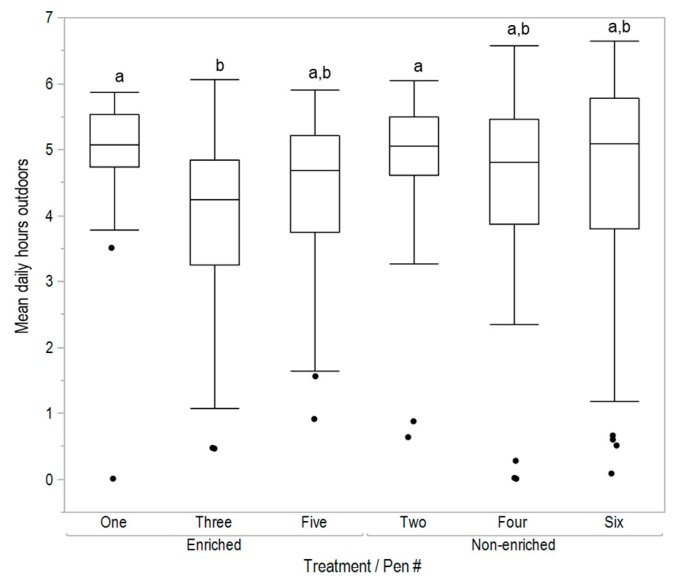
The mean number of hours spent outdoors by hens in each pen within the early rearing treatments (enriched, non-enriched). Data were from 36 to 38 weeks of age; dissimilar letters indicate differences between pens within treatments (*p* < 0.008).

**Figure 4:**
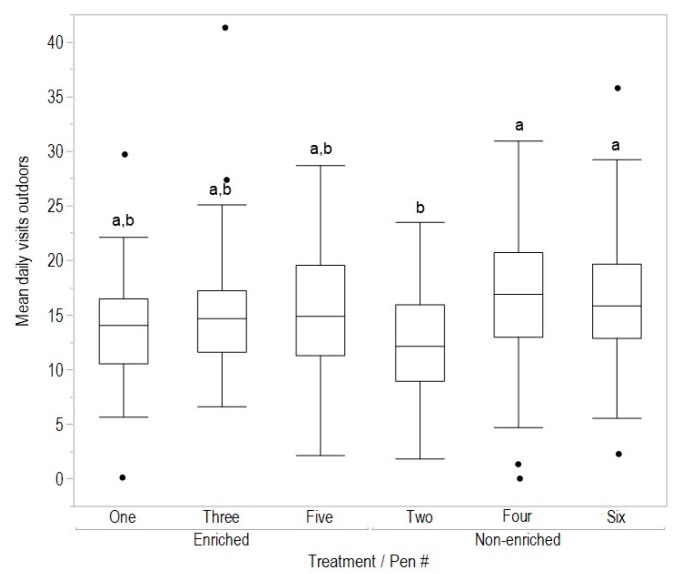
The mean number of daily visits outdoors by hens in each pen within the early rearing treatments (enriched, non-enriched). Data were from 36 to 38 weeks of age; dissimilar letters indicate differences between pens within treatments (*p* < 0.008).

**Figure 5:**
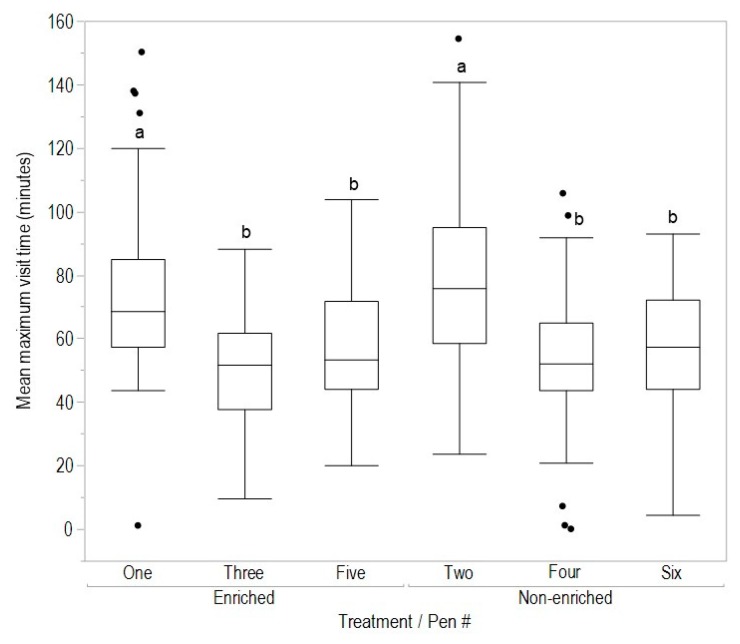
The mean maximum visit time (minutes) outdoors by hens in each pen within the early rearing treatments (enriched, non-enriched). Data were from 36 to 38 weeks of age; dissimilar letters indicate differences between pens within treatments (*p* < 0.008).

**Figure 6:**
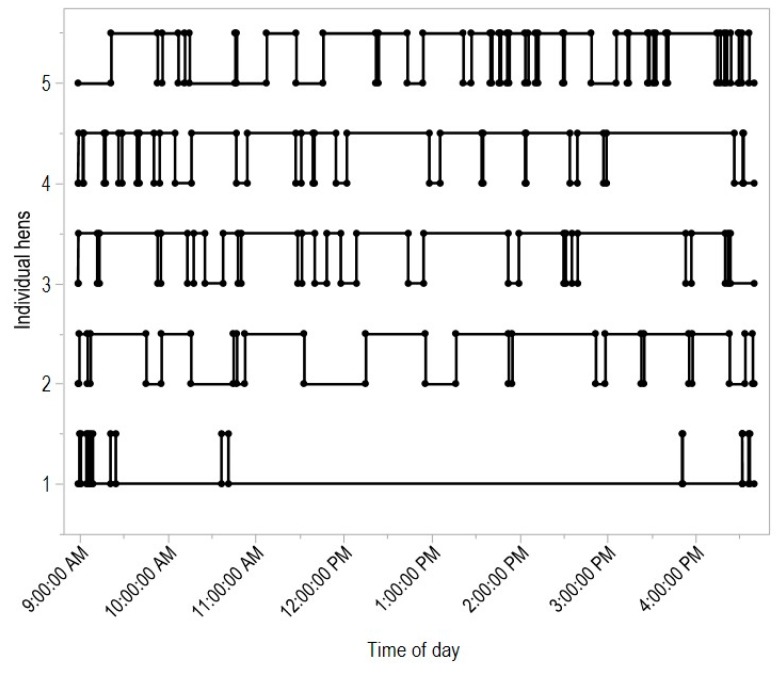
Examples of individual hen movements representing the 5th, 15th, 25th, 35th and 45th most movements during the day for a single pen (non-enriched). A high position indicates that the hen is outside at that time and low position that the hen is inside.

There was no effect of early enrichment rearing treatment on the mean number of hours spent outdoors (F_(1,278)_ = 1.56, *p* = 0.21), daily number of visits to the range (F_(1,278)_ = 0.23, *p* = 0.63) or mean maximum visit time (F_(1,278)_ = 0.15, *p* = 0.70, Figure 2, Figure 3 and Figure 4). However, for all variables there were significant differences between pens within rearing treatments (hours outdoors: F_(4,278)_ = 3.73, *p* = 0.006, Figure 3; visits outdoors: F_(4,278)_ = 3.88, *p* = 0.004, Figure 4; maximum visit time: F_(4,278)_ = 19.59, *p* < 0.001, Figure 5). There was also clear individual variation across all range use measures (Figure 3, Figure 4 and Figure 5). An example of the variation in normal movement patterns of individual hens is shown in Figure 6, which indicates the location (in or out) during the day, of five hens from one pen on a single day. The hens were chosen as representing the 5th, 15th, 25th, 35th and 45th most movements during the day for pen 6 (non-enriched).

### 3.2. Pop-Hole-Following Behaviour

**Figure 7:**
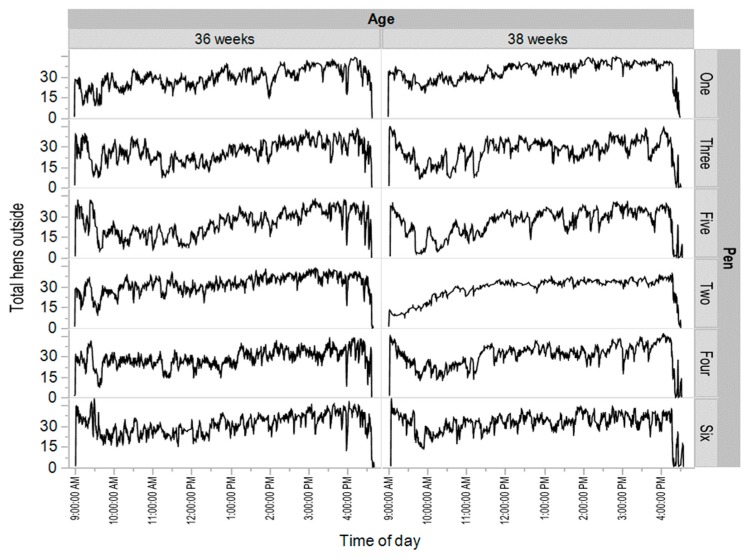
Number of hens outside after each hen movement indoors or outdoors across two days at the start of 36 and 38 weeks of age for each pen of birds (pens one, three and five were from the enriched rearing treatment and pens two, four and six were from the non-enriched rearing treatment).

The actual movements across two single days selected at the beginning of 36 and 38 weeks of age for each pen of birds showed that the mean number of hens outside at any time was consistent over long periods (Figure 7). However, the hen movements suggest that after any hen had moved inside or outside, the movement of the next hen in the group was more likely to be in the same direction, rather than the opposite direction (Figure 7). This would not be the case if hen movements were only to maintain a preferred hen density inside or outside. This was confirmed by analysis of all hen movements in all pens over the period of study, where 67.6% (standard deviation 5.0%) of movements were in the same direction as the previous movement. Only one hen (of 279) had less than 50% of movements following the same direction and less than 2% of the hens had less than 57% following movements.

There were three hens (all in different pens), out of 279 that did not exhibit pop-hole-following behaviour (more than 3SDs below the mean) with pop-hole-following of 46.3%, 52.2% and 52.3%. These values all deviated further from the mean pop-hole-following behaviour than expected from the variation over all hens. Two of them may have been moving at random, without regard to following behaviour, since they were very close to 50%. However, the hen with 46.3% pop-hole-following may have been choosing to move against the normal flow. This hen was of a relatively lower body weight but had no other visible signs of illness (see [8] for more details on regular basic health assessments made on all hens). The highest rate of pop-hole-following behaviour for a single bird was 78.9%, but this was inside the range expected based on the mean and standard deviation of all hens. No distinct ‘leader’ hens were identified within the pens. Hens that consistently started a movement in the other direction would have a pop-hole-following behaviour of less than 50%, because they would frequently oppose the movement of other hens. Only one hen was below 50% and this was not far enough below to conclude it was not just a random occurrence.

The significance of pop-hole-following behaviour was examined by comparison to the data in which all hen movements were random (but based on the mean and standard deviation of the time of movements for each individual hen). The random data ensured that pop-hole-following behaviour did not occur (pop-hole-following movements = 50.5%). It would be expected to be slightly greater than 50% because almost all of the early movements and last movements of each day must be following movements. When the random data were adjusted to include following behaviour (the follower data), there were approximately 65% following movements using a lag time of 40 s. This time interval was determined by increasing or decreasing the interval until the follower data had the same level of pop-hole-following as the actual hens. The 40 s lag interval ha the effect that if a hen may be going to move in the next 40 s then it is more likely to move immediately if a hen moves in the planned direction, but may delay its intended move by up to this interval if a hen moves in the opposing direction. The follower data therefore included general following behaviour without any hen following any other specific hen. This indicated that hens tended to follow other hens when moving inside and outside.

#### 3.2.1. Early Enrichment Treatment Effects on Pop-Hole-Following

There was a significant effect of rearing treatment on the pop-hole-following scores (F_(1,277)_ = 6.93, *p* < 0.009) with the enriched birds showing more following than the non-enriched birds (mean ± SEM raw values: enriched birds 0.71 ± 0.005, non-enriched birds 0.69 ± 0.005). There were also differences between pens of birds within treatment groups (F_(4,277)_ = 11.46, *p* < 0.001).

#### 3.2.2. Associations between Pairs of Hens When Pop-Hole-Following

The actual number of pairs of hens moving together when the set distance between connected hens was 8 or 9 other individuals was not greater than the number of pairs indicated by the random and follower data, in which no hens were paired (Table 2). There was one pair of hens connected using a distance of seven hens, but none closer than this. Therefore, the pairs that appeared as connected were due to random hen movement. Associations moving in and associations moving out were both similar to the results found with the random and follower data.

### 3.3. Associations between Pairs of Hens in Time Together

The results from random data (both ‘random’ and ‘follower’ datasets) showed that an overlap of only 50% in time hens spent together would be expected. However, the actual hens had greater time together than this random value, with a median value greater than the 90th percentile of the random data (Table 3). This association was also consistent over time, based on measurements over six consecutive 10-day periods where the median time together was greater than 50% for all pens at all time periods examined (Table 4). However, there was an increase in time together from a mean of 53.3% in the first period across all pens to 55.4% in the last period across all pens, suggesting a strengthening of the relationships over time (Table 4).

The correlation of time together for each individual pair of hens in each of the six time periods was used to test whether the percentage of time together was consistent for pairs of hens. All correlations were significant (*p* < 0.05) and the correlations for adjacent 10-day periods were significantly greater than the correlation with non-adjacent 10-day periods except for pen 4 (non-enriched, Table 5). This indicates that the relationships between pairs of hens changed over time, but slowly as adjacent periods showed higher correlations (Table 5). However, there was still a significant correlation between the first and last 10-day periods, confirming considerable stability in these pairings.

#### Early Enrichment Treatment Effects on Hen Association

There was a significant effect of rearing treatment on the pen-level percentage of association between pairs of birds (F_(1,30)_ = 21.86, *p* < 0.0001) with the enriched birds showing higher association than the non-enriched birds (mean ± SEM enriched: 55.5 ± 0.60, non-enriched: 53.46 ± 0.45). There were also differences between pens of birds within treatment groups (F_(4,30)_ = 17.48, *p* < 0.0001).

## 4. Discussion

This study analysed RFID data of hen movement in and out of pop-holes in an experimental free-range system to show evidence of pop-hole-following behaviour among individuals within the group and associations between individual hens in simultaneous time spent indoors or outdoors. The extent of both pop-hole-following and hen association varied between the pens. Enriched rearing environments during the first 3 weeks of development also increased both pop-hole-following and levels of hen-pair association. These results have implications for understanding the behaviour of the hen group and potential methods for modifying group dynamics.

Individual birds within each pen varied greatly in their indoor and outdoor movements during the day. In addition to this individual variation, each flock also functioned at the group level with hens exhibiting pop-hole-following behaviour when moving between the indoor and outdoor areas. The extent of pop-hole-following also varied between the pens within rearing treatments. The comparisons with random data indicated that this was not to maintain a specific density, but was indicative of the group acting cohesively. Figure 6 shows the frequency of the indoor/outdoor transitions throughout the day, suggesting that these movements are not just based on circadian rhythms but the dynamic of the group. The movement could be resource-based. Laying hens in their natural environments [14] and other laboratory studies show that hens will aggregate in response to available resources (e.g., water, food) rather than specifically in relation to social cohesion [21,29]. In the free-range system, the outdoor environment may be viewed as a resource for hens that enables foraging and dust bathing [30] and the indoor pen provides perches, food, and water. Thus, individual hens may initially be moving in relation to resource attraction and behavioural activity transitions [31], with the ‘following’ behaviour of other hens socially driven, similar to social facilitation of feeding and dustbathing [32,33]. The pop-hole-following movement could be a measure of how cohesive the group is. However, the implications of this group cohesion are currently unclear. The birds used in this study were kept until 38 weeks of age at which point they were subjected to two stressful events (see [8]), and then the overall experiment was terminated. The birds at this age were still in visibly good condition with minimal feather damage or other welfare concerns (see [8]). Thus, it is unknown whether the group cohesion may have positive (or negative) impacts on hen welfare. Across the flock cycle, a more cohesive group may have better synchronised behaviour such as resting. They may also be less likely to develop aggression and feather pecking if cohesion reduces hen stress, which is associated with increased feather pecking [26]. Alternatively, there may be higher competition for resources at specific times. Such hypotheses remain to be tested but such experiments may result in a greater understanding of the group entity in free-range and other non-cage laying hen housing systems.

Hens exhibited clear following of other hens in or out of the pen but they did not appear to follow any specific hens. In their natural environments, jungle fowl will form hierarchies in small groups [15] and in experimental tests with small numbers of birds (n = 10), hens will recognise specific individuals [16,34]. However, as group size increases, the formation of a hierarchy is thought to dissipate when it becomes too costly to establish dominance with continuously unfamiliar individuals [16]. Thus, the group sizes used in the current study may not have enabled clear flock hierarchies. At an even larger scale as for commercial layer systems, it is unknown if similar following patterns may exist or if individual hens interact with each other in a different manner when any individual recognition is unlikely. Hen movement between indoors and outdoors may not be influenced by social rank even if this was able to be established—an avenue for future research in small hen groups.

Hens did not appear to follow other specific hens when moving inside or outside but they did spend more time simultaneously indoors or outdoors with certain other hens. On average, pairs of hens appeared together 55% of the time, with 10% of pairs together 58% or more of the time. Although this proportion of time together is not high, it was significant by being several standard deviations outside the range when using random movements, and it was stable over time. These percentage associations were solely based on hens being simultaneously present either indoors or outdoors; no further data were collected on proximity of specific hens and thus the results must be interpreted with caution. Hens in aviary systems have individual patterns of locational preferences throughout the day and thus specific hens may spend time together if they consistently both visit certain areas [35,36]. Whether locational preferences of one hen result from the locational preferences of another hen they may associate with remains to be assessed. Further study with wireless sensors [37] could remotely monitor preferential proximity by hen pairs for complete assessment of the presence of social networks and how this may influence range use in free-range systems.

Enrichment in the first three weeks of life increased the degree of pop-hole-following behaviour and hen-pair association in time simultaneously spent inside or outside. This suggests that the enriched birds formed a more socially-cohesive flock than the non-enriched birds. Although it must be noted that all enriched and non-enriched birds were reared in a single pen per treatment prior to being separated into six pens, thus an effect of room regardless of enrichment treatment may have played a role. There have been few prior studies assessing the effects of physical enrichment on subsequent social dynamics of laying hens. Early enrichment has been shown to reduce fear responses of domestic chicks [38] and also reduce responses to stressful events in adult broilers [39]. For these birds, the larger overall study [8] showed that the enriched birds had reduced albumen corticosterone stress responses when their range area was intentionally reduced, accompanied by higher behavioural change in their ranging patterns [8]. The early environment is important for social development of laying hens [40]. Provision of enrichments may either allow young birds to better control their social interactions by providing areas of escape, or may increase affiliative behaviour by providing preferred resources to congregate around (e.g., chicks were observed resting together underneath a plastic tree that was provided as one of the enrichments). Future experiments and detailed observations of birds during the rearing period may confirm these hypotheses. The potential for early environmental enrichment to impact the flock dynamics of adult layers warrants further investigation.

## 5. Conclusions

Analysis of RFID data of individually-tagged hens in a free-range system showed that birds behaved as a cohesive flock in their use of indoor and outdoor areas with movement patterns through the pop-holes influenced by other birds (i.e., not random). Birds provided with early enrichment showed improved social cohesion but further study is required to replicate these results and document any long-term welfare impacts of differences in flock-level movement patterns and cohesiveness. Analyses of ranging patterns in larger commercial flocks would be valuable in understanding group behavioural differences.

## Figures and Tables

**Table 1:** Mean ± SD of the pen values used to generate the random individual-bird dataset ^1^.

Measure	One	Two	Three	Four	Five	Six
Mean1	1.98 ± 0.24	1.95 ± 0.34	1.68 ± 0.44	2.08 ± 0.32	1.37 ± 0.50	1.49 ± 0.47
SD1	1.05 ± 0.19	1.04 ± 0.19	0.86 ± 0.20	1.02 ± 0.16	0.59 ± 0.22	0.69 ± 0.16
Mean4	2.22 ± 0.22	2.22 ± 0.22	2.24 ± 0.34	2.26 ± 0.38	2.25 ± 0.42	2.28 ± 0.47
SD4	0.71 ± 0.15	0.67 ± 0.18	0.65 ± 0.15	0.71 ± 0.16	0.67 ± 0.14	0.62 ± 0.18
MeanOut	2.86 ± 0.17	2.89 ± 0.20	2.81 ± 0.24	2.79 ± 0.21	2.74 ± 0.22	2.70 ± 0.29
SDOut	0.55 ± 0.07	0.56 ± 0.08	0.51 ± 0.06	0.52 ± 0.07	0.51 ± 0.05	0.53 ± 0.04
MeanIn	2.51 ± 0.18	2.42 ± 0.20	2.69 ± 0.21	2.55 ± 0.23	2.59 ± 0.25	2.51 ± 0.20
SDIn	0.54 ± 0.08	0.55 ± 0.09	0.52 ± 0.09	0.49 ± 0.07	0.50 ± 0.07	0.51 ± 0.13

^1^ Mean1 is the logarithm (base 10) of the mean number of seconds between when the pop holes were first opened and the hen first moved outside. Mean4 is the logarithm of the mean number of seconds between the last movement inside for that day and 16:30 h, when the pop holes were closed. MeanOut is the logarithm of the mean number of seconds outside on each visit, i.e., log (total time outside/number of movements outside). MeanIn is the logarithm of the mean number of seconds inside on each visit, i.e., log (total time inside/number of movements inside). SD1, SD4, SDOut, SDIn are the standard deviations of those means for individual hens.

**Table 2:** Mean number (±SEM) of pairs of hens detected as possible social pairs moving in or out with a median separation distance of no more than 8 or 9 hens in between ^1^.

Data	9 Hens	8 Hens
In		
Actual	13.7 ± 4.9	1.83 ± 1.0
Follower	17.8	1.83
Random	18.7	2.67
Out		
Actual	12.8 ± 4.9	2.00 ± 1.0
Follower	16.7	2.00
Random	19.5	2.33

^1^ Standard error for the ‘follower’ and ‘random’ data was very small across the combined runs. In a single run, the SEM was similar to the SEM of the actual data as the data were created using actual means and SEM.

**Table 3:** Median and 90th percentile of time that hen pairs spent together (both inside and outside).

Group	Median Overlap	90th Percentile
Actual hens	54.5%	57.0%
Random Group	49.2%	50.4%
Follower group	49.3%	50.5%

**Table 4:** Median percentage of time together inside and outside for all possible pairs of hens during successive 10-day periods.

Treatment	Pen	Period 1	Period 2	Period 3	Period 4	Period 5	Period 6
Enriched	One	53.0	52.0	54.2	53.3	53.2	54.0
Non-enriched	Two	51.6	51.8	53.6	53.4	51.6	52.3
Enriched	Three	53.3	53.9	54.9	54.5	54.8	58.4
Non-enriched	Four	51.4	51.6	52.3	51.6	52.1	55.1
Enriched	Five	55.7	58.3	57.8	60.4	59.5	57.8
Non-enriched	Six	54.4	56.0	57.1	56.4	55.2	54.8

**Table 5:** Mean ± SEM of the correlational *R*-values between adjacent and non-adjacent 10-day periods that were assessed for the percentage of association between all possible pairs of hens ^1^.

Treatment	Group	Adjacent Periods	Non-Adjacent Periods	*p*-Value
Enriched	One	0.423 ± 0.031	0.314 ± 0.020	0.003
Non-enriched	Two	0.299 ± 0.045	0.187 ± 0.015	0.019
Enriched	Three	0.333 ± 0.028	0.314 ± 0.016	0.54
Non-enriched	Four	0.222 ± 0.011	0.168 ± 0.019	0.013
Enriched	Five	0.545 ± 0.026	0.457 ± 0.014	0.003
Non-enriched	Six	0.471 ± 0.009	0.405 ± 0.018	0.001

^1^*p*-values indicate the significance of the difference between adjacent and non-adjacent periods. All *R*-values were significantly greater than zero (*p* < 0.001).

## Supplementary Materials

The following are available online at http://www.mdpi.com/2076-2615/8/11/210/s1, Materials 1: Generation of the random dataset.
