# Supplementary material for: Using Radio-Frequency Identification Technology to Measure Synchronised Ranging of Free-Range Laying Hens
Source: Animals (Basel). 2018 Nov 16;8(11):210. doi: 10.3390/ani8110210 (PMC6262442; doi:10.3390/ani8110210)
Supplement: Supplementary file 1 [file animals-08-00210-s001.zip › animals-371720-supplementary.docx]

Supplementary Material 1: Generation of the random dataset

Mean1 = logarithm of the mean time between when the pop holes were first opened and the hen first moved outside. SD1 is the standard deviation of this log time.

MeanOut = logarithm of the mean time outside on each visit i.e. log(total time outside/number of movements outside). SDOut is the standard deviation of this log time.

MeanIn = logarithm of the mean time inside on each visit i.e. log(total time inside/number of movements inside). SDIn is the standard deviation of this log time. The last movement of the day inside was not included, since the hens are forced to remain inside overnight.

Mean4 = logarithm of the time between the last movement inside for that day and 1630 h, when the pop holes were closed. SD4 is the standard deviation of this log time.

RandG(Mean,SD) is a function used in Delphi Pascal to set a random number with a normal distribution of the specified mean and standard distribution.

For each hen (h = 1 to 50) set the time of the first movement for that day as minutes after 0900 h

T1_h_ = 10^(RandG(Mean1,SD1));

For each hen (h = 1 to 50) set the time of the last movement for that day as minutes before 1630 h.

TL_h_ = 10^(RandG(Mean4,SD4));

Repeat

{ Section for Follower data only

Repeat

Find the hen with next movement. If the movement of that hen would be in the opposite direction to the most recent hen movement than add 40 seconds to the next movement time for that hen.

Until the next hen movement is in the same direction, or 40 seconds have expired since the last movement, or the next movement is for a hen that has already had a 40 second delay applied.

End of extra section for Follower data }

Find the hen with the next movement. If the hen is inside, it moves out, if it is outside it moves inside.

Reset the next movement for that hen.
If the hen is now inside,
then Tout_h_ = 10^(RandG(MeanIn,SDIn))
otherwise the hen is outside,
so TIn_h_ = 10^(RandG(MeanOut,SDOut))
If the next movement by that hen would occur later than TL_h_ then the hen will remain inside if already in, but if outside it will move inside at TL_h_.

Until all hens are inside after their last movement for the day.
